# The Efficacy of Pharmacological Interventions in the Treatment of Major Depressive Disorder and Bipolar Depression With Mixed Features: A Systematic Review

**DOI:** 10.1111/bdi.70049

**Published:** 2025-08-13

**Authors:** Naomi Xiao, Liyang Yin, Serene Lee, Kayla M. Teopiz, Sabrina Wong, Gia Han Le, Sebastian Badulescu, Heidi Ka. Ying. Lo, Maj Vinberg, Bing Cao, Iria Grande, Joshua D. Rosenblat, Roger S. McIntyre

**Affiliations:** ^1^ Brain and Cognition Discovery Foundation Toronto Canada; ^2^ Department of Health Sciences Queen's University Kingston Canada; ^3^ Department of Pharmacology and Toxicology University of Toronto Canada; ^4^ Mood Disorder Psychopharmacology Unit University Health Network Toronto Canada; ^5^ Institute of Medical Science University of Toronto Toronto Canada; ^6^ Department of Psychiatry School of Clinical Medicine, LKS Faculty of Medicine, the University of Hong Kong Hong Kong; ^7^ The Early Multimodular Prevention and Intervention Research Institution (EMPIRI), Mental Health Centre, Northern Zealand, Copenhagen University Hospital – Mental Health Services CPH Copenhagen Denmark; ^8^ Department of Clinical Medicine Faculty of Health and Medical Sciences, University of Copenhagen; ^9^ Key Laboratory of Cognition and Personality, Faculty of Psychology, Ministry of Education, Southwest University Chongqing P. R. China; ^10^ Departament de Medicina Facultat de Medicina i Ciències de la Salut, Universitat de Barcelona (UB) Barcelona Spain; ^11^ Bipolar and Depressive Disorders Unit, Hospital Clinic de Barcelona. c. Villarroel Barcelona Spain; ^12^ Institut D'investigacions Biomèdiques August Pi i Sunyer (IDIBAPS), c. Villarroel Barcelona Spain; ^13^ Institute of Neurosciences (UBNeuro) Barcelona Spain; ^14^ Centro de Investigación Biomédica en red de Salud Mental (CIBERSAM), Instituto de Salud Carlos III Madrid Spain; ^15^ Department of Psychiatry University of Toronto Toronto Canada

**Keywords:** atypical antipsychotics, bipolar disorder, depression, major depressive disorder, mixed features, mixed features specifier, suicidality

## Abstract

**Background:**

There is a need to provide up‐to‐date, clinically translatable data as it relates to the treatment of a major depressive episode (MDE) with mixed features.

**Methods:**

PubMed and OVID were searched from inception to July 22, 2024. Randomized controlled trials (RCTs) investigating the efficacy of pharmacological agents for adults with bipolar disorder (BD) or major depressive disorder (MDD) in an MDE with mixed features were included. Risk of bias was assessed using the Cochrane Risk of Bias Tool for Randomized Studies (RoB2).

**Results:**

A total of seven studies were included in this systematic review. The studies identified were all short‐term acute studies ranging from 6 to 8 weeks. Treatment with lurasidone, olanzapine, cariprazine, lumateperone, quetiapine, and ziprasidone was associated with statistically significant reduction of depressive symptoms in MDEs with mixed features. Only lumateperone is studied in both BD subtypes [bipolar I disorder (BD‐I), bipolar II disorder (BD‐II)] and MDD, wherein efficacy in mixed features was the prespecified primary outcome. Lurasidone has a single study in MDD, while ziprasidone has data in a mixed sample of BD‐II and MDD. Data for the other agents in mixed features is post hoc. Co‐occurring hypomanic symptoms generally improved, and there was no significant difference between the above treatments and placebo with respect to hypomanic symptom severity intensification or treatment‐emergent affective switching.

**Conclusion:**

Select atypical antipsychotics are effective in alleviating depressive symptoms in persons with mixed features; albeit, much of the data is obtained from post hoc analysis. Minimal evidence exists for the efficacy of lithium or valproate in the treatment of depressive episodes with mixed features. Antidepressant monotherapy has not been adequately evaluated in depressive episodes with mixed features. In addition, there is a pressing need for a consistent definition of mixed presentations to guide future interventional studies.

## 1 Introduction

Mixed features specifier applies to either a hypomanic, manic, or major depressive episode (MDE) and is defined and operationalized in the Diagnostics and Statistical Manual of Mental Disorders, Fifth Edition Text Revision (DSM‐5‐TR) as three or more non‐overlapping “opposite polarity” symptoms [1]. In addition to the aforementioned DSM‐5‐TR definition of mixed features, other definitions of mixed have been proposed that define mixed features with different severities of depressive and hypomanic symptoms [2, 3, 4, 5, 6]. The predominance of depressive symptoms and episodes in bipolar disorder (BD) and their defining feature in major depressive disorder (MDD) indicates that a major depressive episode (MDE) with mixed features is a common presentation in clinical practice [7, 8, 9]. Convergent evidence indicates that mixed features proxy a more complex illness presentation (e.g., severity, suicidality, non‐recovery, chronicity, recurrence, and comorbidity) [10, 11]. In addition, the presence of mixed features contributes to misdiagnosis and delayed diagnosis in persons with BD [6, 7, 9, 12].

Separately, it has been reported that conventional antidepressants are suboptimally effective in the treatment of MDEs with mixed features [13]. Additionally, individuals living with MDEs and mixed features exhibit greater susceptibility to treatment‐related affective destabilization [14, 15]. Taken together, there is an urgent need to identify safer and more effective treatment strategies for persons experiencing MDEs with mixed features as part of BD or MDD. Herein, we conducted a systematic review of randomized controlled trials (RCTs) that sought to determine the efficacy of a pharmacological intervention in the treatment of adults with bipolar I disorder (BD‐I), bipolar II disorder (BD‐II), or MDD presenting with an MDE and mixed features.

## 2 Methods

### 2.1 Search and Selection Strategy

In accordance with the Preferred Reporting Items for Systematic Review and Meta‐Analyses (PRISMA) guidelines, a systematic search was conducted on PubMed and OVID (MedLine, Embase, AMED, PsychINFO, and JBI EBP) from inception to July 22, 2024 [16]. In addition, the reference lists of the obtained articles were manually searched. The pharmacological agents included in the search terms were lithium salts, antidepressants, typical or atypical antipsychotics, and anticonvulsants that are US Food and Drug Administration (FDA)‐approved to treat MDD or BD. The following Boolean logic search string was used to search the databases: (“Amitriptyline” OR “Citalopram” OR “Clomipramine” OR “Fluvoxamine” OR “Mirtazapine” OR “Nortriptyline” OR “Paroxetine” OR “Phenelzine” OR “Desipramine” OR “Duloxetine” OR “Escitalopram” OR “Sertraline” OR “Venlafaxine” OR “Agomelatine” OR “Desvenlafaxine” OR “Gepirone” OR “Levomilnacipran” OR “Moclobemide” OR “Selegiline” OR “Tranylcypromine” OR “Vilazodone” OR “Vortioxetine” OR “Bupropion” OR “Dextromethorphan” OR “Dextromethorphan‐bupropion” OR “Ketamine” OR “Esketamine” OR “Fluoxetine” OR “Zuranolone” OR “Cariprazine” OR “Lurasidone” OR “Quetiapine” OR “Olanzapine” OR “Lumateperone” OR “Aripiprazole” OR “Asenapine” OR “Haloperidol” OR “Paliperidone” OR “Risperidone” OR “Ziprasidone” OR “Iloperidone” OR “Clozapine” OR “Amisulpride” OR “Brexpiprazole” OR “Carbamazepine” OR “Valproate” OR “Valproic Acid” OR “Divalproex” OR “Gabapentin” OR “Oxcarbazepine” OR “Pregabalin” OR “Topiramate” OR “Lamotrigine” OR “Lithium”) AND (“Major Depressive Disorder” OR “MDD” OR “Depression” OR “Depressive Episode” OR “Bipolar Disorder” OR “Bipolar I Disorder” OR “Bipolar II Disorder” OR “BD‐I” OR “BD‐II” OR “Bipolar Depression” OR “Bipolar I Depression” OR “Bipolar II Depression”) AND (“Mixed Features” OR “Mixed Features Specifier” OR “Mixed Episodes” OR “Mixed States” OR “Depressive Mixed Episodes”).

After removing duplicate studies, two reviewers (NX, LY) independently screened the article titles and abstracts for relevance against the eligibility criteria (Table 1) and resolved all discrepancies via discussion. The same protocol was followed for full‐text screening.

**TABLE 1:** Eligibility criteria.

Inclusion Criteria	Randomized controlled trials or post hoc analyses Acute studies Depression must be measured using at least one of the following assessments: MADRS, HAM‐D, PHQ9, CUDOS, QIDS‐SR, YMRS Participants must be ≥ the age of 18 Participants must have a primary diagnosis of major depressive disorder (MDD) bipolar depression (BD) according to the Diagnostic and Statistical Manual of Mental Disorders (DSM) Fourth Edition (DSM‐IV), Fourth Edition Text Revision (DSM‐IV‐TR), Fifth Edition (DSM‐5), or Fifth Edition Text Revision (DSM‐5‐TR) Participants must have mixed features. Mixed features can be defined as an MDE with 2 or more hypo/manic symptoms, or an MDE and a YMRS score ≥ 4 Participants must be administered with a lithium salt, antidepressant, typical or atypical antipsychotic, or anticonvulsant that is US Food and Drug Administration (FDA)‐approved to treat MDD or BD. Studies reporting on multiple states (depressed with mixed features, manic, hypomanic, etc.) must present data for each state separately English language Full‐text article available online
Exclusion Criteria	Secondary articles (e.g., systematic reviews, narrative reviews, meta‐analyses, drug profiles, guidelines, protocols, and theses) Case reports, case series, cohort studies, cross‐sectional studies, observational studies, chart reviews Maintenance studies (excluded due to an absence of maintenance studies evaluating treatment in persons with mixed features) Studies that only observe physiological measures (e.g., EEG) Participants below the age of 18 Primary diagnosis other than MDD or BD. No treatment/administration with an antidepressant, lithium salt, typical or atypical antipsychotic, or anticonvulsant that is US Food and Drug Administration (FDA)‐approved to treat MDD or BD. Co‐administration with another drug outside the list of FDA‐approved antidepressants, antipsychotics, anticonvulsants, or lithium salts. Animal studies. Full‐text was not available online.

### 2.2 Data Extraction

Data extraction of the included studies was conducted by one reviewer (NX) using a piloted data extraction template. The information to be extracted was established a priori and included: (1) author(s) and publication year, (2) study design, (3) sample size, (4) definition of mixed features used, (5) dosage, (6) study assessment(s), and (7) outcome(s) of interest (Table 2).

**TABLE 2:** Summary of the included randomized controlled trials of Pharmacological Interventions in the Treatment of Major Depressive Disorder and Bipolar Depression with Mixed Features.

Author(s)	Study design	Sample size	Pharmacological agent(s)	Dosing regimen	Study assessment(s)	Outcome(s) of interest
**Major depressive disorder**						
Suppes et al. (2016) [17]	Randomized, double‐blind, placebo‐controlled study	211 total participants	Lurasidone (*n* = 109) Placebo (*n* = 100)	20 or 40 mg/day lurasidone for 6 weeks	MADRS, YMRS, SDS, CGI‐S, HAMA	Lurasidone treatment was associated with significantly more significant decreases in MADRS total score from baseline to week 6 and a higher percentage of responders and remitters than placebo. The lurasidone group had greater decreases in YMRS, SDS, and CGI‐S scores than placebo.
**Bipolar depression**						
Benazzi et al. (2009) [18]	Post hoc analysis of a double‐blind placebo‐controlled study	833 total participants Mixed (*n* = 376) Non mixed (*n* = 457)	Olanzapine (*n* = 173) Olanzapine and fluoxetine (*n* = 37) Placebo (*n* = 166)	5–20 mg/day olanzapine, or 6 and 25, 6 and 50, or 12 and 50 mg/day olanzapine and fluoxetine for 8 weeks	MADRS, YMRS, CGI‐S	Both combined olanzapine and fluoxetine treatment and olanzapine monotherapy showed significantly higher response rates than placebo.
McIntyre et al. (2015) [7]	Post hoc analysis of a double‐blind, placebo‐controlled study	485 total participants Mixed (*n* = 272) Non mixed (*n* = 213)	Lurasidone (*n* = 323) Placebo (*n* = 162)	20–60 mg/day or 80–120 mg/day lurasidone for 6 weeks)	MADRS, CGI‐BP‐S, YMRS, QIDS‐SR, SDS	Lurasidone treatment was associated with significantly greater decreases in MADRS and CGI‐BP‐S scores, and significantly higher response and remission rates than placebo. QIDS‐SR and SDS scores decreased significantly from baseline to endpoint in the lurasidone group but not the placebo group. YMRS scores did not change significantly in either group.
McIntyre et al. (2019) [9]	Post hoc analysis of 3 randomized, double‐blind, placebo‐controlled trials	1383 total participants Mixed (*n* = 808) Non mixed (*n* = 575)	Cariprazine (*n* = 923) Placebo (*n* = 460)	0.75, 1.5, or 3 mg/day cariprazine for 6 or 8 weeks	MADRS, YMRS, HAMD_17_, CGI‐S	Mixed patients treated with cariprazine showed significantly greater decreases in MADRS total score and in several MADRS individual items compared to placebo. Both cariprazine dosage groups had greater response and remission rates. Significantly greater improvements in HAMD_17_ and CGI‐S scores over placebo were seen in both cariprazine groups. Decreases in YMRS score of the treatment groups were numerically but not statistically greater than placebo.
McIntyre et al. (2023) [19]	Post hoc analysis of a randomized, double‐blind, placebo‐controlled outpatient study	376 total participants Mixed (*n* = 156) Non mixed (*n* = 220)	Lumateperone (*n* = 188) Placebo (*n* = 188)	42 mg lumateperone for 43 days	MADRS, YMRS, CGI‐BP‐S, Q‐LES‐Q‐SF	In mixed patients, lumateperone treatment was associated with a significantly greater decrease in MADRS and CGI‐BP‐S scores, and a numerically greater decrease in YMRS score compared to placebo.
Wang et al. (2023) [20]	Randomized pilot trial	56 total participants	Quetiapine monotherapy (*n* = 36) Quetiapine + valproate (*n* = 11) Quetiapine + lithium (*n* = 10)	300–600 mg/day (quetiapine monotherapy) for 2 weeks, followed by either continued quetiapine monotherapy, 600–900 mg/day (quetiapine + lithium), or 1000 mg/day (quetiapine + valproate) for 6 weeks	MADRS, YMRS, CUDOS‐M	There were no significant differences in changes of MADRS, YMRS, or CUDOS scores between the combined treatment groups. Quetiapine + lithium showed a significant decrease in MADRS and YMRS scores. Quetiapine + valproate showed a significant reduction in MADRS score, but not in YMRS or CUDOS scores. Quetiapine monotherapy showed a consistent improvement in all measures.
**Major depressive disorder and bipolar depression**						
Patkar et al. (2012) [21]	Randomized, double‐blind, placebo‐controlled study	72 total participants MDD (*n* = 29) BD (*n* = 43)	Ziprasidone (*n* = 35) Placebo (*n* = 38)	80–160 mg/day ziprasidone for 6 weeks	MADRS, YMRS, MRS, GAF, CGI‐BP	Treatment with ziprasidone was associated with a significantly greater decrease in MADRS score, a higher response rate, and a higher remission rate than placebo. MRS scores did not change significantly in either group.

Abbreviations: BD, Bipolar Depression; CGI‐BP‐S, Clinical Global Impression Bipolar—Severity of Illness; CGI‐S, Clinical Global Impression—Severity of Illness; CUDOS‐M, Clinically Useful Depression Outcome Scale supplemented with questions for the DSM‐5 mixed features specifier; HAMA, Hamilton Anxiety Rating Scale; HAMD_17_, Hamilton Depression Rating Scale (17 items); MADRS, Montgomery–Åsberg Depression Rating Scale; MDD, Major Depressive Disorder; QIDS‐SR, Quick Inventory of Depressive Symptomatology—Self Report; Q‐LES‐Q‐SF, Quality of Life Enjoyment and Satisfaction Questionnaire—Short Form; SDS, Sheehan Disability Scale; YMRS, Young Mania Rating Scale.

### 2.3 Quality Assessment

Two independent reviewers (NX and LY) used the Cochrane Risk of Bias Tool for Randomized Studies (RoB2) to assess the included RCTs' risk of bias (Table 3). All conflicts were resolved via discussion.

**TABLE 3:** Quality assessment of the included studies using the Cochrane Risk of Bias Tool for Randomized Studies (RoB2).

Study	Item						Quality rating
	1	2	3	4	5	6	
Benazzi et al. (2009) [18]	L	L	L	L	M	L	Good
Patkar et al. (2012) [21]	L	M	L	L	L	L	Good
McIntyre et al. (2015) [22]	L	L	L	L	L	L	Good
Suppes et al. (2016) [17]	L	L	L	L	L	L	Good
McIntyre et al. (2019) [9]	L	L	L	L	L	L	Good
McIntyre et al. (2023) [19]	L	L	L	L	L	L	Good
Wang et al. (2023) [20]	L	L	L	L	L	L	Good

Abbreviations: H, high risk of bias; L, low risk of bias; M, medium risk of bias.

## 3 Results

### 3.1 Search Results

**FIGURE 1:**
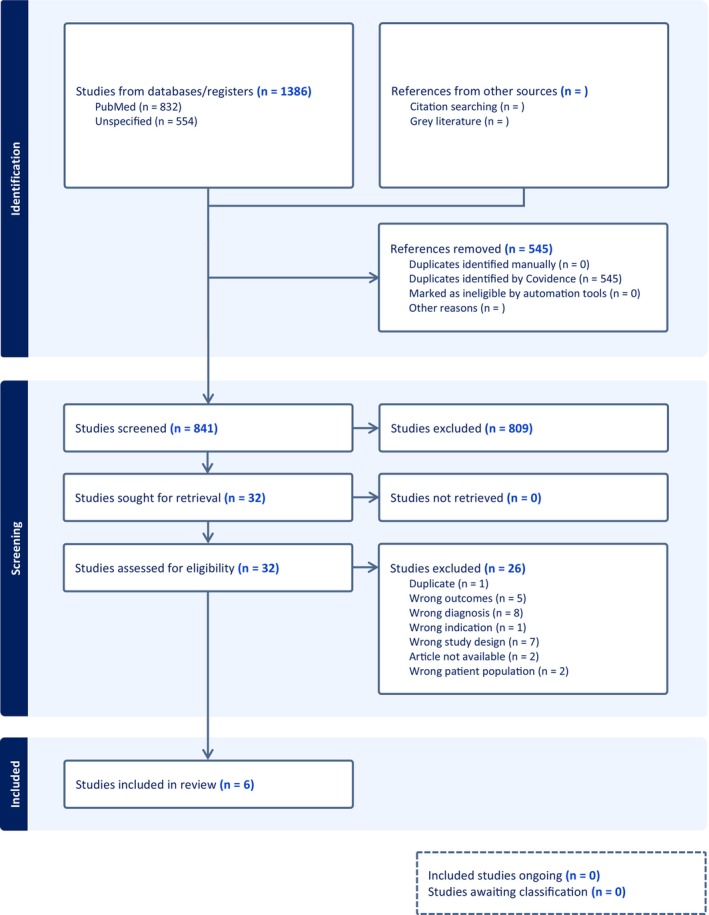
PRISMA flow diagram of literature selection.

The database search identified 1386 studies; 545 duplicates were removed. The remaining 841 studies were screened by titles and abstracts, of which 33 articles were retrieved for full‐text screening against the eligibility criteria. Following the full‐text screening, 26 studies were excluded for various reasons described in Figure 1. A total of seven studies were included in this systematic review.

### 3.2 Methodological Quality

All included studies reported whether the allocation of participants to experimental groups was randomized and whether researchers and participants were blinded to treatment allocation. Many studies did not have a pre‐specified primary outcome to evaluate the effect of a treatment for an MDE with mixed features. Instead, many included studies only evaluated participants with mixed features via post hoc analysis. Furthermore, the identified studies had no universal definition of mixed features.

Regarding quality assessment, none of the included studies had a high overall risk of bias, and most studies had a low risk of bias for all six items (Table 3). The risk of bias of the study conducted by Benazzi et al. [18] was determined to be of medium risk in item 5 because the Clinical Global Impression—Severity of Illness (CGI‐S) scores were assessed but not reported. The study conducted by Patkar et al. [21] had a medium risk of bias in item 2 as the integrity of the double‐blind procedure was not sufficiently reported.

### 3.3 Efficacy of Pharmacological Treatment in Major Depressive Disorder Patients With Mixed Features

We identified a single study that evaluated the efficacy of pharmacological treatment in MDD with mixed features. Suppes et al. [17] conducted a randomized, double‐blind, placebo‐controlled study assessing the effect of lurasidone 20–40 mg/day (*n* = 109) compared to placebo (*n* = 100) in patients with MDD and mixed features (defined as having two or more manic symptoms) over a 6‐week period. Efficacy measures included the Montgomery–Åsberg Depression Rating Scale (MADRS), the Young Mania Rating Scale (YMRS), the Sheehan Disability Scale (SDS), CGI‐S, and the Hamilton Anxiety Rating Scale (HAM‐A) [17].

It was reported that a significantly greater least squares (LS) mean change from baseline to week 6 in MADRS total score was observed for the lurasidone‐treated group compared to placebo (–20.5 and –13.0 respectively; *p* < 0.001; effect size, 0.80) [17]. The percentage of patients that met a priori response criteria at week 6 (≥ 50% reduction from baseline in MADRS score) was 64.8% for the lurasidone group vs. 30.0% for the placebo group [*p* < 0.001; Number Needed to Treat (NNT) = 3, Last Observation Carried Forward (LOCF)] [17]. The percentage of patients that met a priori remission criteria at week 6 (MADRS score ≤ 12) was 49.1% for the lurasidone group vs. 23.0% for the placebo group (*p* < 0.001; NNT = 4 [LOCF]) [17].

There was a significantly greater LS mean change from baseline to week 6 in CGI‐S score for the lurasidone‐treated patients compared to the placebo group (−1.8 and −1.2, respectively; *p* < 0.001; effect size, 0.60) [17]. The lurasidone group showed statistical superiority compared to placebo in CGI‐S score from week 2 to week 6 [17].

The lurasidone‐treated group showed a significantly greater least squares mean change in SDS score than the placebo group (–11.2 vs. –6.4; *p* < 0.001; LOCF) [17]. The LS mean change in YMRS score was also significantly greater at week 6 for the lurasidone group than the placebo group (–7.0 vs. –4.9; *p* < 0.001; LOCF) [17].

### 3.4 Efficacy of Pharmacological Treatment in Bipolar Depression Patients With Mixed Features

A total of five RCTs evaluating the effect of pharmacological treatment in persons with BD and mixed features were identified.

#### 3.4.1 Lumateperone

McIntyre et al. [19] conducted a post hoc analysis of a phase 3, randomized, double‐blind, placebo‐controlled outpatient study assessing the efficacy of 42 mg/day lumateperone or placebo for 43 days for persons with BD‐I and BD‐II. Of the 376 participants, 156 had mixed features (defined as having a baseline YMRS score ≥ 4) and 220 were nonmixed. The efficacy measures used were MADRS, YMRS, Clinical Global Impression; Bipolar—Severity of Illness (CGI‐BP‐S), and the Quality of Life Enjoyment and Satisfaction Questionnaire—Short Form (Q‐LES‐Q‐SF) [19].

In patients with mixed features, lumateperone treatment was associated with a significantly greater LS mean change in MADRS total score from baseline to day 43 compared to placebo [Least Squares Mean Difference (LSMD), −4.4; 95% CI, −7.26 to −1.52; effect size, −0.52; *p* < 0.01] [19]. Within the mixed population, the lumateperone group had a significantly greater decrease in CGI‐BP‐S total score from baseline to endpoint compared to placebo (LSMD, −0.7; 95% CI, −1.43 to −0.05; effect size, −0.37; *p* < 0.05) [19]. There was also a statistically significant improvement in LS mean Q‐LES‐Q‐SF percent score at day 43 in the lumateperone group compared to placebo (LSMD, 5.9; 95% CI, 1.09–10.71; effect size, 0.41; *p* < 0.05) [19]. There was no statistically significant increase or decrease in total YMRS scores in persons receiving lumateperone at the endpoint [19]. Mania, recorded as a treatment‐emergent adverse event (TEAE), was identified in a similar percentage of persons receiving lumateperone or placebo (1.4% and 2.4%, respectively) [19].

#### 3.4.2 Cariprazine

A post hoc analysis performed by McIntyre et al. [23] pooled the data of three randomized, double‐blind, placebo‐controlled studies (MD‐56, MD‐53, and MD‐54) evaluating the efficacy of cariprazine treatment in BD‐I patients with mixed features. Of the 1383 participants, 808 were classified as mixed (defined as having a baseline YMRS score ≥ 4). Participants received 0.75 mg/day, 1.5 mg/day, or 3 mg/day cariprazine for 6 or 8 weeks. The MADRS, YMRS, 17‐item Hamilton Depression Rating Scale (HAMD_17_), and CGI‐S were used as study assessments [23].

In mixed patients, significantly greater improvements in MADRS total score were seen in the cariprazine group compared to placebo within the first 2 weeks and persisting until week 6. In the 3 mg/day cariprazine group, there was a significant improvement over placebo starting at week 1 (*p* < 0.05) [23]. For all cariprazine dosage groups, the LSMD of cariprazine versus placebo was statistically significant in favor of cariprazine at week 6: the 1.5 mg/day cariprazine group had an LSMD of –2.51 ± 0.85 (*p* < 0.01) and the 3 mg/day group had an LSMD of –2.86 ± 0.86 (*p* < 0.001) [23]. Both doses of cariprazine demonstrated significantly greater efficacy than placebo in several MADRS individual items. The 1.5 mg/day cariprazine group showed greater LS mean changes in the apparent sadness (*p* < 0.05 vs. placebo), reported sadness (*p* < 0.01), inner tension (*p* < 0.05), reduced appetite (*p* < 0.05), concentration difficulties (*p* < 0.01), and lassitude (*p* < 0.05) items [23]. The 3 mg/day cariprazine group showed greater LS mean changes in the apparent sadness (*p* < 0.001), reported sadness (*p* < 0.01), reduced appetite (*p* < 0.01), concentration difficulties (*p* < 0.05), and lassitude (*p* < 0.05) items [23]. In all cariprazine groups, the percentage of mixed patients who met the MADRS response and remission criteria at week 6 was significantly higher than placebo [23]. The NNT for response was 12 in the 1.5 mg/day group and 9 in the 3 mg/day group, and the NNT for remission was 10 in both groups compared to placebo (all, *p* < 0.05) [23].

Significantly greater improvements over placebo in HAMD_17_ scores were seen for both cariprazine groups beginning after week 2 and persisting to week 6. The HAMD_17_ LSMD compared to placebo at week 6 was –1.86 ± 0.65 (*p* < 0.01) for the 1.5 mg/day group and –1.55 ± 0.67 (*p* < 0.05) for the 3 mg/day group [23]. The differences between cariprazine dosage groups in HAMD_17_ total score change at week 6 were not statistically significant (LSMD = –0.3108; *p* = 0.6350) [23]. There was a significantly greater percentage of patients who met the HAMD_17_ remission criteria at week 6 in the 1.5 mg/day cariprazine group compared to placebo (NNT = 7; *p* < 0.01). In the 3 mg/day cariprazine group, this percentage was numerically but not significantly greater than placebo [23].

Both cariprazine groups showed significantly greater improvements in CGI‐S score compared to placebo within the first 2 weeks and continuing to week 6. The LSMD of the treatment groups compared to placebo was –0.24 ± 0.10 (*p* < 0.05) for the 1.5 mg/day group and –0.25 ± 0.10 (*p* < 0.10) for the 3 mg/day group [23]. Differences between cariprazine doses in CGI‐S score change from baseline to week 6 were not significant (LSMD = 0.0027; *p* = 0.9789) [23]. There was also a significantly higher percentage of patients who met the CGI‐S remission criteria at week 6 in both cariprazine groups compared to placebo [23].

Although YMRS total scores decreased in all treatment groups with higher cariprazine doses showing greater decreases (placebo = −1.36; 1.5 mg/day = −1.56; 3 mg/day = −1.73), the differences between groups were not statistically significant [23]. Differences between cariprazine doses in YMRS total score change at week 6 were not statistically significant (LSMD = 0.1690; *p* = 0.5395) [23]. Rates of treatment‐emergent mania were numerically but not significantly lower in both cariprazine groups compared to placebo [23].

#### 3.4.3 Quetiapine, Valproate, and Lithium

In a randomized pilot trial conducted by Wang et al. [20], 56 participants with BD and mixed features (defined as 2 or 3 manic symptoms during an MDE) received quetiapine monotherapy for 2 weeks. They received 100 mg on day 1, with 100 mg increased each day until day 3. The dose range was then adjusted to 300–600 mg/day. The participants who responded (MADRS score reduction ≥ 20%) to quetiapine monotherapy continued their treatment for another 6 weeks [20]. Those who did not respond sufficiently (MADRS score reduction < 20%) were randomly assigned to either quetiapine combined with valproate or quetiapine combined with lithium for 6 weeks [20]. The quetiapine + lithium group received 300 mg on day 1, then increased to 600–900 mg/day within 3 days. The quetiapine + valproate group started at 500 mg/day, and the dose was adjusted to 1000 mg/day within 3–5 days [20]. Study assessments included MADRS, YMRS, Clinically Useful Depression Outcome Scale supplemented with questions for the DSM‐5 mixed features specifier (CUDOS‐M), and HAMA [20].

No significant differences in changes of MADRS, YMRS, or CUDOS scores were observed between the quetiapine + valproate group and the quetiapine + lithium group between baseline and week 8 [20]. The quetiapine + lithium group showed a significant reduction of mean MADRS (–7.18, *p* = 0.025) and YMRS (–4.82, *p* = 0.047) scores from baseline to week 8. All measures at each visit were lower than the previous visit [20]. For the quetiapine + valproate group, a significant reduction was only observed in mean MADRS (–8.6, *p* = 0.027) score between baseline and week 8. Although the MADRS score had an overall decrease in this group, it increased between the second and third visits [20]. The YMRS score of the quetiapine + valproate group decreased slightly from week 2 to week 4, then increased at week 8, and the CUDOS scores decreased at each visit [20]. In the quetiapine monotherapy group, all measures at each visit were lower than the previous visit, and there was a lower point estimation in YMRS and CUDOS scores compared to the other two groups [20].

#### 3.4.4 Lurasidone

A post hoc analysis of a double‐blind, placebo‐controlled study was conducted by McIntyre et al. [22] evaluating the efficacy of lurasidone treatment in 485 participants with BD‐I, of which 272 had mixed features (defined as having a YMRS score ≥ 4). The lurasidone dosage groups were 20–60 mg/day (starting dose: 20 mg/day for 7 days) and 80–120 mg/day (20 mg/day on days 1–2, 40 mg/day on days 3–4, 60 mg/day on days 5–6, and 80 mg/day on day 7) [22]. After day 7, doses were adjusted to optimize efficacy and tolerability. The study assessments included MADRS, CGI‐BP‐S, YMRS, QIDS‐SR, SDS, and Q‐LES‐Q‐SF [22].

Lurasidone treatment was associated with a significantly greater LS mean change in MADRS total score from baseline to week 6 compared to placebo (–15.7 vs. –10.9; *p* = 0.001; effect size, 0.48) [22]. Superiority in MADRS score compared to placebo was seen in the lurasidone group from week 2 to endpoint. The differences between lurasidone dosage groups were nonsignificant (*p* = 0.127) [22]. There was a significantly greater percentage of responders (≥ 50% reduction from baseline in MADRS total at LOCF endpoint) in the lurasidone group compared to placebo (51.1% vs. 32.2%; *p* = 0.003; NNT = 6; LOCF endpoint) [22]. Similarly, the percentage of remitters (MADRS total ≤ 12 at LOCF endpoint) was significantly greater in the lurasidone group than the placebo group (39.6% vs. 24.4%; *p* = 0.014; NNT = 7; LOCF endpoint) [22].

The lurasidone group had a significantly greater LS mean change in the CGI‐BP‐S depression score than placebo (−1.9 vs. −1.2; *p* < 0.001; effect size, 0.57) [22]. Superiority in the CGI‐BP‐S depression score compared to placebo was observed from week 2 to the endpoint. Treatment with lurasidone was associated with a Quick Inventory of Depressive Symptomatology—Self Report (QIDS‐SR) LS mean change from baseline to endpoint of –7.2 ± 0.4 (*p* < 0.01) while the placebo group had a numerical but not significant change of –4.9 ± 0.6 [22]. The LS mean change in SDS from baseline to endpoint was –9.3 ± 0.9 for the lurasidone group (*p* < 0.01) and –5.2 ± 1.2 for the placebo group (not significant) [22]. The LS mean change in YMRS from baseline to week 6 was non‐significant for both the lurasidone and placebo groups [22].

#### 3.4.5 Combination of Olanzapine and Fluoxetine

Benazzi et al. [18] performed a post hoc analysis of a double‐blind, placebo‐controlled study assessing the effect of olanzapine monotherapy and olanzapine in combination with fluoxetine (OFC) in persons with BD‐I and mixed features. Of the 833 total participants with BD, 376 had mixed features (defined as two or more hypo/manic symptoms during an MDE) [18]. The mixed patients were randomly assigned to receive placebo (*n* = 166), 5–20 mg/day olanzapine (*n* = 173), or 6 and 25, 6 and 50, or 12 and 50 mg/day of olanzapine and fluoxetine (*n* = 37) for 8 weeks [18]. MADRS, YMRS, and CGI‐S scores were assessed [18].

The OFC treatment group had 16/37 (43.2%) responders [defined as a ≥ 50% reduction in total MADRS score and < 2 concurrent hypo/manic symptoms (measured by YMRS) after the treatment period] [18]. 46/173 (26.6%) of the participants taking olanzapine responded, and 27/166 (16.3%) of the placebo group responded [18]. When compared to placebo, both combined treatment (OR = 3.91; 95% CI, 1.80–8.49; *p* = 0.0006) and olanzapine monotherapy (OR = 1.95; 95% CI, 1.14–3.34; *p* = 0.014) showed significantly higher response rates [18]. There were significantly lower dropout rates in the combined treatment group (29.7%) compared to olanzapine monotherapy (53.8%) and placebo (59.6%) (olanzapine monotherapy vs. combined treatment: OR = 2.67; 95% CI, 1.23–5.75; *p* = 0.12; placebo vs. combined treatment: OR = 3.48; 95% CI, 1.61–7.54; *p* = 0.002) [18]. There was no significant difference in dropout rates between the olanzapine and placebo groups (OR = 1.30; 95% CI, 0.84–2.01; *p* = 0.227) [18]. The switch rates to mania/hypomania of each group were 8.5% (7/82) for combined treatment, 6.8% (24/351) for olanzapine, and 7.9% (28/355) for placebo (*χ* ^2^ = 0.426, df = 2, *p* = 0.808) [18].

### 3.5 Efficacy of Pharmacological Treatment in Major Depressive Disorder and Bipolar Depression With Mixed Features

Two RCTs evaluating the effect of pharmacological treatment in persons with MDD and BD with mixed features were identified.

#### 3.5.1 Ziprasidone

In a randomized, double‐blind, placebo‐controlled study conducted by Patkar et al. [21], 72 participants with either MDD (*n* = 29) or BD‐II (*n* = 43) received ziprasidone or placebo for 6 weeks. All participants were diagnosed with the DSM‐IV criteria for an MDE and met 2 or 3 symptoms of DSM‐IV‐defined mania [21]. The initial dose for the ziprasidone group was 40 mg/day, which was then increased by 20–40 mg/day weekly up to a dose of 80–160 mg/day for the remainder of the treatment period [21]. Study assessments included the MADRS, YMRS, CGI‐BP, the Mania Rating Scale (MRS), and the Global Assessment of Functioning scale (GAF) [21]. Ziprasidone treatment was associated with a very significant decrease in mean MADRS score from baseline to endpoint (23.4 ± 6.5 to 12.0 ± 10.9, *p* = 0.0038) [21]. The placebo group showed a change in mean MADRS score from 25.1 ± 7.9 at baseline to 19.2 ± 9.3 at endpoint, and the outcome change difference between ziprasidone and placebo [95% CI] was 5.4 [0.6, 10.2] (*p* < 0.05) [21]. The differences in MADRS score between ziprasidone and placebo were significant at week 3 (*p* < 0.001), week 5 (*p* < 0.05), and week 6 (*p* < 0.05) [21]. The treatment response (defined as 50% improvement in MADRS and MRS) rate was 52.9% for ziprasidone versus 28.9% for placebo (*χ* ^2^ = 4.29, df = 1, *p* = 0.04) [21]. The treatment remission (defined as MADRS score ≤ 9 and YMRS score ≤ 11) rate was 50.0% for ziprasidone versus 18.4% for placebo (*χ* ^2^ = 8.05, df = 1, *p* = 0.0045) [21]. The severity of the MRS scores did not significantly change from baseline to endpoint in either group [21].

#### 3.5.2 Lumateperone

A separate study conducted by Durgam et al. [24, 25] evaluated lumateperone 42 mg monotherapy compared to placebo in adults with either MDD or BD‐I/BD‐II meeting DSM‐5 criteria for an MDE and mixed features specifier. This is the first study that has pre‐specified a depression outcome in persons who a priori enrolled on the basis of having mixed features. Lumateperone 42 mg once daily resulted in a statistically significant and clinically meaningful reduction in MADRS total score compared to placebo at week 6 (i.e., combined MDD and BD with mixed features: LSMD = −22125.7, effect size = 0.64, *p* < 0.0001; MDD with mixed features: LSMD = −5.9; effect size = 0.67, *p* < 0.0001; BD with mixed features LSMD = −5.7; effect size = 0.64, *p* < 0.0001) [25].

## 4 Discussion

Herein, we identified seven studies that met the inclusion criteria for this systematic review. Of the seven reports, most used post hoc analysis to determine the efficacy and safety of a pharmacological treatment as the prespecified and primary efficacy outcome. No single agent was found to be efficacious as part of two large adequately powered double‐blind placebo‐controlled trials wherein evaluating outcomes in persons enrolled primarily on the basis of mixed features as part of an MDE (often employed as the benchmark of demonstrated efficacy). Second‐generation antipsychotics were the class of drugs most studied, notably lurasidone, lumateperone, cariprazine, olanzapine, and quetiapine. Available evidence also indicates that each of the aforementioned interventions did not result in a significantly higher rate of treatment‐emergent affective switching. Moreover, lithium, valproate, and lamotrigine, three agents commonly prescribed for persons living with bipolar disorder, have been insufficiently evaluated in adults with depressive episodes with mixed features. The inadequacy of the evidence base for these three agents does not negate the possibility that they may be meaningfully effective in depressive episodes with mixed features.

Mixed features have historical, conceptual, nosologic, and therapeutic implications. Mixed features, states, and episodes have been variably defined and described for over a century [10]. Supplanting mixed episodes with mixed features in the DSM‐5 in 2013 was a pivot towards a more dimensional and unifying conceptualization of mixed features across mood disorders [5]. Debate continues about the essential aspects of mixed features and whether the exclusion of non‐overlapping symptoms may potentially reduce sensitivity in the interest of specificity [26, 27]. Debate also continues regarding the central features of mixed and whether mixed features identify a unique sub‐population and/or are a transitory phenomenon [28, 29]. Separate lines of observation also suggest that mixed features may be increasingly replacing euphoric presentations perhaps due to prescription behavior (e.g., antidepressant utilization), social and economic determinants, and/or increased rates of comorbidity (e.g., obesity) [30]. Against this background, there is an urgent need to identify safe and effective therapeutic avenues for MDEs with mixed features, which are estimated to affect 25%–40% of all adults presenting mood disorders clinically [7].

The methodological quality of the included studies was generally robust, with most studies exhibiting a low risk of bias across multiple domains. However, the variability in definitions of mixed features and the predominantly post hoc nature of many analyses suggest a need for more standardized and prospective research designs. Furthermore, there are an inadequate number of studies for any agent to definitively conclude that any particular agent(s) are uniquely efficacious in the treatment of MDEs with mixed features. In addition, our analysis is constrained to only those subpopulations within the full enrolled population who met criteria for mixed features specifier (with the exception of the single study evaluating lumateperone that prespecified efficacy in persons with BD‐I, BD‐II and MDD). There is an absence of large, replicated, adequately controlled studies that sought to determine the efficacy and safety of most interventions in the treatment of MDEs with mixed features. Further research should aim to standardize the criteria for mixed features by adopting the DSM‐5‐TR's mixed features specifier more uniformly.

In addition, most of the available studies identified were post hoc analyses of a primary study that was principally designed to evaluate the efficacy and safety of an intervention in BD. There are relatively few studies that prespecified depression outcomes as the primary efficacy variable in persons presenting with an MDE with mixed features. The advantages of post hoc analysis are pragmatism and convenience; however, unequivocal statements of efficacy cannot be made on post hoc analyses alone [31]. Furthermore, each of the studies evaluating the efficacy of atypical antipsychotics in mixed features was sponsored by a pharmaceutical company that has marketing authorization for the product, which introduces a bias that needs to be considered when interpreting the findings. In addition, all of the studies we identified were short in duration (e.g., 6 weeks) and there is a need for long‐term efficacy studies evaluating interventions in persons with mixed features, as short duration studies may not be adequate to fully assess acute response. We delimited the inclusion of studies that were acute, as there are no long‐term controlled studies primarily evaluating interventions in the treatment of MDEs with mixed features during the maintenance phase of bipolar or unipolar disorder. Long‐term efficacy is critical to discern, as persons with mixed presentations have a greater susceptibility to relapse and recurrence [32]. Mixed features may also predict the diagnostic transition from MDD to BD, which may favor the use of psychotropics with mood‐stabilizing properties. In addition, multiple definitions of mixed features were employed across the included studies, which may affect generalizability and validity.

The significant reductions in depressive symptoms of participants in the included studies have important clinical implications for treating patients with mixed features during MDEs. Given the complexities of mixed presentations, individual responses to select pharmacological agents vary, highlighting the need for a personalized, data‐driven approach to treatment. This would enhance our clinical practice, particularly for individuals who do not respond adequately to standard mood stabilizers or antidepressants.

In summary, an evidence gap exists concerning decision support in the treatment and management of adults presenting with an MDE with mixed features as part of either BD or MDD. Notwithstanding the high prevalence, severity, chronicity, and hazards posed by mixed features presentations, relatively few studies primarily evaluate this population. Nevertheless, expert consensus exists that second‐generation atypical antipsychotics with efficacy in the treatment of MDEs may be preferred agents in persons presenting with mixed features highly linked to emotional instability [6, 33, 34]. Consensus also exists that there is a need to investigate other innovative strategies including but not limited to lithium, glutamate modulators (e.g., ketamine), selective Kappa opioid receptor antagonists, KCNQ modulators, and orexin antagonists [35]. By advancing our understanding of the efficacy and limitations of these pharmacological agents, we can better tailor treatment strategies for individuals with mixed features during MDEs, ultimately enhancing their health outcomes. Further research with more standardized diagnostic definitions and prospective research designs is needed in this field.

## Conflicts of Interest

Dr. Roger S. McIntyre has received research grant support from CIHR/GACD/National Natural Science Foundation of China (NSFC) and the Milken Institute; speaker/consultation fees from Lundbeck, Janssen, Johnson & Johnson, Alkermes, Neumora Therapeutics, Boehringer Ingelheim, Sage, Mitsubishi Tanabe, Purdue, Pfizer, Otsuka, Takeda, MindMed, Neurocrine, Neurawell, Supernus, Bausch Health, Axsome, Novo Nordisk, Kris, Sanofi, Eisai, Intra‐Cellular, NewBridge Pharmaceuticals, Viatris, Abbvie, and Atai Life Sciences. Dr. S. Roger McIntyre is the CEO of Braxia Scientific Corp. Iria Grande has received grants and has served as a consultant, advisor, or CME speaker for the following entities: ADAMED, Angelini, Casen Recordati, Esteve, Ferrer, Gedeon Richter, Janssen Cilag, Lundbeck, Lundbeck‐Otsuka, Luye, SEI Healthcare, and Viatris outside the submitted work. She also receives royalties from Oxford University Press, Elsevier, and Editorial Médica Panamericana. Kayla M. Teopiz has received fees from Braxia Scientific Corp. All other co‐authors (Naomi Xiao, Liyang Yin, Angela T.H. Kwan, Gia Han Le, Sabrina Wong, Kyle Valentino, Hayun Choi, and Serene Lee) authors declare no conflicts of interest.

## Data Availability

The data that support the findings of this study are available in PubMed at https://pubmed.ncbi.nlm.nih.gov/ and in OVID at https://ovidsp.ovid.com/.
